# Long‐Term Clinical, Radiographic and Esthetic Outcomes of Zirconia Dental Implants: A 10‐Year Prospective Multicenter Study

**DOI:** 10.1111/clr.70089

**Published:** 2026-01-22

**Authors:** S. Roehling, K. H. Bormann, M. M. Bornstein, S. Laval, F. Thieringer, M. Gahlert

**Affiliations:** ^1^ Clinic for Oral and Cranio‐Maxillofacial Surgery, University Hospital Basel, Swiss MAM Research Group, Department of Biomedical Engineering University of Basel Basel Switzerland; ^2^ Private Dental Clinic “Oralchirurgie T1” PD Dr. Gahlert & PD Dr. Roehling Munich Germany; ^3^ Clinic for Oral‐ and Maxillofacial Surgery Hannover Medical School Hannover Germany; ^4^ Dental Clinic “Oralchirurgie am Hafen” Hamburg Germany; ^5^ Department of Oral Health & Medicine University Center for Dental Medicine Basel UZB, University of Basel Basel Switzerland; ^6^ Clinic for Oral‐ and Maxillofacial Surgery Katharinenhospital, Klinikum Stuttgart Stuttgart Germany; ^7^ Sigmund Freud Private University Vienna Austria

**Keywords:** ceramic implants, clinical investigation, dental implants, implant survival, marginal bone loss, success rate, survival rate, yttria stabilized tetragonal zirconia, zirconium oxide

## Abstract

**Objectives:**

This study aimed to prospectively investigate the long‐term clinical performance of a commercially available one‐piece zirconia dental implant system over 10 years.

**Material and Methods:**

A multicenter, open‐label study was conducted at three clinical centers in Germany. Forty‐four patients with single‐tooth gaps meeting specific inclusion criteria received 44 yttria‐stabilized zirconia (Y‐TZP) implants featuring a sandblasted and acid‐etched (ZLA) surface. Clinical and radiographic follow‐ups were performed at 1, 3, 5, and 10 years to assess implant survival, success, peri‐implant bone levels, and esthetic outcomes using Pink Esthetic Score (PES) and White Esthetic Score (WES).

**Results:**

At the 10‐year follow‐up, 35 patients with 35 implants were available for evaluation. The estimated 10‐year survival rate was 97.7% (95% CI: 97.27–98.13). Three implants (8.6%) showed biological complications, including peri‐implant mucositis in 2 implants (5.7%) and peri‐implantitis in 1 implant (2.9%), leading to a success rate of 91.4% (95% CI: 76.9–97.8). Peri‐implant bone loss was moderate, averaging 1.20 (±0.61) mm over 10 years, with stable bone levels observed after the initial remodeling phase. Esthetic outcomes revealed a slight increase in PES (7.4 to 7.8) and a minor decrease in WES (7.0 to 6.7) between years 5 and 10.

**Conclusions:**

Zirconia implants demonstrated high long‐term survival and success rates, moderate bone loss, and favorable esthetic outcomes. These findings support their use as a clinically reliable and esthetically acceptable long‐term alternative to titanium implants. However, the limited sample size highlights the need for further confirmation in larger cohorts.

## 1 Introduction

Currently, a wide variety of dental implant types are available on the market. The differences between these implants can be characterized not only by design—such as conical versus parallel implant bodies or aggressive versus conventional threads—but also by the materials used, namely titanium versus ceramic. Consequently, when selecting an implant design and material, it is essential to consider not only anatomical and functional indications but also the individual needs and preferences of patients. Currently, titanium implants are considered the gold standard in implant dentistry (Albrektsson et al. 2008). Their long‐term clinical reliability has been well documented in numerous studies, with reported survival rates ranging from 95.1% to 98.8% for titanium implants after 10 years (Buser et al. 2012; Di Francesco et al. 2022; Fischer and Stenberg 2012; Kim et al. 2018).

However, the choice of implant material has been critically discussed and has become the focus of some interest in the literature in recent years. Although titanium implants have demonstrated excellent long‐term outcomes, some studies have raised concerns about their potential—albeit infrequently seen—to trigger hypersensitivity or inflammatory reactions. Furthermore, associations have been reported between plaque accumulation, biocorrosion, and the presence of titanium particles in the surrounding tissues, which may contribute to biological complications such as peri‐implantitis (Chen et al. 2023; Franchi et al. 2004; Mombelli et al. 2018; Suárez‐López Del Amo et al. 2018).

Zirconium dioxide (zirconia, ZrO_2_) has emerged as an alternative implant material on the market. Compared to other ceramics, zirconia offers superior biomechanical properties, enabling these implants to withstand oral occlusal forces (Christel et al. 1989; Kohal et al. 2015). Over the past two decades, manufacturing processes have been refined to enhance the clinical performance and biomechanical stability of zirconia implants. This technical evolution is reflected in the different generations of zirconia implants, which demonstrate improved clinical performance (Roehling et al. 2018). The latest generation of zirconia implants exhibits predictable osseointegration (Gahlert et al. 2012; Roehling et al. 2019) and high clinical survival rates comparable to those of conventionally used titanium implants (Balmer et al. 2020; Gahlert et al. 2022; Kiechle et al. 2023; Kohal et al. 2020). Furthermore, randomized clinical trials have confirmed comparable results for zirconia and titanium implants in terms of implant survival, marginal bone stability, and esthetic outcomes over follow‐up periods of up to 80 months (Koller et al. 2020; Ruiz Henao et al. 2024).

Systematic reviews on the clinical performance of the latest generation of zirconia implants have estimated mean survival rates ranging from 95% to 97.2% after 1 and 2 years (Pieralli et al. 2017; Roehling et al. 2018) and 97.2% after 5 years of functional loading (Roehling et al. 2023).

Despite promising short‐ and medium‐term outcomes, only a limited number of studies have investigated commercially available zirconia implants with follow‐up periods extending up to 10 years. The reported survival rates in these investigations vary considerably, ranging from 93.4% to 100% (Borgonovo et al. 2021; Brunello et al. 2022), which highlights the need for additional evidence on their long‐term clinical performance. In particular, prospective investigations with comprehensive evaluation of functional, biological, and esthetic aspects are essential to better understand the performance of zirconia implants over extended periods of functional loading. Against this background, the present study represents the first prospective multicenter investigation to evaluate the clinical, radiographic, and esthetic outcomes of a commercially available zirconia implant system over a 10‐year follow‐up period.

## 2 Material and Methods

This study was designed as a prospective, open‐label, single‐arm clinical multicenter investigation conducted in accordance with the principles outlined in the Declaration of Helsinki in 2013, in accordance with ISO14155 and following the Strengthening the Reporting of Observational Studies in Epidemiology (STROBE) guidelines for reporting observational studies (von Elm et al. 2014). Patients were treated at three clinical centers in Germany: a private dental practice with special focus in implant dentistry in Munich, the Clinic for Oral and Maxillofacial Surgery at Hannover Medical School, and the Clinic for Oral and Maxillofacial Surgery at Katharinenhospital in Stuttgart. The research protocol and consent form were approved by the ethics committee of each participating institution (ethical approval number 011/1616), and informed consent was obtained from all patients. The study is registered at www.clinicaltrials.gov (study no. NCT02163395). Results from the 1‐year, 3‐year, and 5‐year follow‐ups have already been published (Bormann et al. 2018; Gahlert et al. 2022, 2016). This paper presents data collected after 10 years of functional loading of this patient cohort.

### 2.1 Patient Population

Patient eligibility was verified against predefined criteria. The main inclusion criteria were male or female patients aged at least 18 years with single‐tooth gaps in the mandible or maxilla at tooth positions 16 to 26 or 36 to 46 (according to FDI classification). Additionally, the neighboring teeth at the implant sites had to be naturally healthy, and the implant sites must have been healed for at least 8 weeks post‐extraction. For cases with insufficient bone volume, bone augmentation procedures were required to be performed either at least 12 weeks prior to or simultaneously with implant placement.

Patients not meeting the inclusion criteria were excluded from the study. The following exclusion criteria were also established:

- Uncontrolled diabetes
- Mucosal diseases
- Untreated periodontitis or gingivitis
- Endodontic lesions
- Smoking more than 10 cigarettes per day
- Probing pocket depth of at least 4 mm on neighboring teeth
- Inadequate oral hygiene
- Severe bruxism
- History of local radiotherapy

Furthermore, patients were excluded from the study if primary implant stability could not be achieved during surgery or if the implants could not be placed in a suitable prosthetically driven position.

### 2.2 Implants

In the present study, patients were treated with a monotype (1‐piece) full ceramic yttria‐stabilized zirconia (Y‐TZP) implant (Straumann PURE Ceramic Implant, Straumann Group, Basel, Switzerland). These implants featured a sandblasted, large‐grit, and acid‐etched surface (ZLA). The monotype design integrated the abutments directly into the implant body, offering two different abutment heights: 4 mm and 5.5 mm. In addition, the tissue‐level design of this specific implant includes a polished shoulder with a height of 1.8 mm, designed to support soft tissue health and facilitate supra‐crestal positioning. The implants had a diameter of 4.1 mm, with lengths ranging from 8 mm to 12 mm.

### 2.3 Surgical and Restorative Procedure

Implant placement surgeries were conducted under local anesthesia following a standardized protocol. Briefly, implants were placed after the elevation of a mucoperiosteal flap, as flapless surgery was not eligible for this study. Osteotomy preparation and implant placement adhered to the manufacturer's standard instructions. If deemed necessary, simultaneous bone augmentation was performed exclusively using autogenous bone.

The position of the implant shoulder was determined based on the esthetic requirements for tissue‐level implants, typically positioned 1–2 mm below the anticipated mucosal margin of the prosthesis. As a result, the marginal mucosa extended above the implant shoulder, allowing all implants to heal in an unloaded transmucosal position. To prevent soft tissue overgrowth on the implant shoulder, protective caps were placed on the abutments when indicated. In cases where no protective cap was used, the implant shoulder was uncovered as necessary prior to the insertion of the provisional prosthesis.

Sutures were removed one to 2 weeks post‐surgery, with a provisional prosthesis placed between 11 and 13 weeks after implant placement. The final prosthesis was delivered 24 to 28 weeks post‐implantation.

For the crowns, lithium disilicate glass‐ceramics or zirconia ceramics were predominantly used. The crowns were directly cemented onto the implant abutment using permanent glass ionomer luting cement. To prevent excessive cement during the cementation process, the ceramic crowns were designed with a cement‐draining hole on the occlusal side. Crown cementation followed a defined clinical protocol that included a moderate to thin application of cement using a small dental brush. Additionally, any submarginal cement excess was removed with specialized dental floss (Superfloss, Oral B, Procter & Gamble Service GmbH, Schwalbach am Taunus, Germany) during the curing process. The absence of excess cement was clinically and radiographically confirmed. After implant placement and crown insertion, periapical radiographs were obtained to assess the outcomes. For further details, please refer to the 1‐, 2‐ and 5‐year studies involving the same patient cohort (Bormann et al. 2018; Gahlert et al. 2022, 2016).

### 2.4 Clinical Examination

Patients were invited for clinical check‐ups 1, 2, 3, 5, and 10 years after implant placement. During these examinations, the presence of plaque and sulcus bleeding was assessed not only around the implants and neighboring teeth but also recorded per patient, allowing for both a site‐specific and patient‐level evaluation. Additionally, all implants and restorations were photographed at the 5‐ and 10‐year visits.

In addition to the scheduled study follow‐ups, all patients received routine clinical maintenance care twice a year throughout the entire 10‐year observation period. These appointments included clinical assessments as well as professional dental cleanings to support peri‐implant health and overall oral hygiene.

Throughout the observation period, adverse events (AEs) related to the patients' general health—such as changes in medications or the diagnosis of general conditions like heart attacks or diabetes—were closely monitored. Technical complications concerning the implant body and prosthetic restoration, including chipping, fractures, or debonding, were also recorded.

Moreover, the evaluations included inquiries about any complications the patients had experienced since their previous check‐up, such as pain or bleeding. Biological complications, such as peri‐implant soft tissue issues (e.g., swelling, fistulas, and mucositis) and peri‐implantitis, were also monitored to ensure comprehensive assessment and management of potential concerns.

### 2.5 Radiographic Examination

Standard periapical radiography was conducted using individualized, customized radiographic stents at baseline (implant placement), at crown insertion, and at 1, 2, 3, 5, and 10 years post‐implant surgery. This imaging was performed to evaluate marginal peri‐implant bone remodeling throughout the observation period. The digital, standardized periapical radiographs were analyzed by an independent expert using ImageJ software (http://imagej.nih.gov/ij/index.html).

Calibration of each implant was performed using the known distance between the implant threads to adjust for any imaging distortions, allowing linear measurements to be accurately converted into millimeters. Consideration was given to distortions that may arise from changes in the X‐ray relative to the true dimensions of the implants.

The following parameter was evaluated:

- The distance from the implant shoulder to the first visible bone‐to‐implant contact (DIB) at two sites (mesial and distal) of the implant. The margin of the implant shoulder was identified as the transition point from the implant shoulder to the cemented crown. The DIB value for each implant was calculated as the average of the mesial and distal measurements.

For each implant, the DIB was assessed directly after implant placement (DIB_0_), after crown insertion (DIB_0.5_), and at 1 (DIB_1_), 2 (DIB_2_), 3 (DIB_3_), 5 (DIB_5_), and 10 years (DIB_10_) post‐placement. Additionally, the following differences were calculated to quantify the amount of crestal bone remodeling over time: DIB_0_—DIB_0.5_, DIB_0_—DIB_1_, DIB_0_—DIB_2_, DIB_0_—DIB_3_, DIB_0_—DIB_5_, and DIB_0_—DIB_10_.

### 2.6 Esthetic Outcomes

The esthetic outcomes of peri‐implant tissues and crowns were evaluated at 5 and 10 years using the combined Pink Esthetic Score (PES) and White Esthetic Score (WES) (Belser et al. 2009). The PES/WES provides a reproducible method for assessing the aesthetics of peri‐implant tissues surrounding single‐tooth implant crowns. Each score is based on five specific parameters:

PES:

- Mesial papilla
- Distal papilla
- Curvature of the facial mucosa
- Level of the facial mucosa
- Root convexity/soft tissue color and texture at the facial aspect of the implant site

WES:

- General tooth form
- Outline and volume of the clinical crown
- Color, which encompasses hue and value assessments
- Surface texture
- Translucency and characterization

Each variable is rated on a scale of 2‐1‐0, where 2 represents the best score and 0 the poorest. All variables are assessed in comparison to a natural contralateral reference tooth. Clinical photographs of the peri‐implant soft tissues and implant crowns, including at least one natural adjacent tooth on each side, were captured using a digital camera after 5 and 10 years. The PES/WES analyses were conducted by one experienced clinician who was not involved in the surgical or prosthetic treatment of the patients. To minimize bias and ensure reproducibility, evaluations were performed twice on different days, with a 14‐day interval between assessments.

### 2.7 Implant Survival

Implant survival was defined as retention of the implant at the 10‐year follow‐up.

### 2.8 Implant Success

Based on the clinical and radiographic data obtained, each implant was classified as either a success or a failure according to previously published success criteria (Buser et al. 1990). An implant was considered successful if there were no signs of pain, foreign body discomfort, dysesthesia, recurrent peri‐implant infection with suppuration, implant mobility, or continuous peri‐implant radiolucency. Conversely, implant failure was defined as the loss of the implant after insertion. Importantly, the determination of implant success also included the assessment of biological complications such as mucositis and peri‐implantitis. Mucositis was defined as the presence of bleeding and/or suppuration on gentle probing, with or without increased probing depth compared to previous examinations, and without radiographic bone loss beyond the crestal changes associated with initial remodeling. Peri‐implantitis, on the other hand, was diagnosed when bleeding and/or suppuration on probing was accompanied by increased probing depth and radiographic evidence of bone loss beyond the expected crestal remodeling (Hirooka and Renvert 2019). These biological parameters were integrated into the success evaluation to provide a comprehensive assessment of implant performance.

### 2.9 Statistical Analysis

Data management was conducted using DMSys software (Version 5.3), while IBM SPSS statistical software (Version 21, IBM) was utilized for statistical analysis. Descriptive summary statistics were calculated for all documented parameters, including mean values and standard deviations. The analyses included all patients who received an implant and were not lost to follow‐up (full‐analysis population with imputation).

To account for patients lost to follow‐up, implant survival rates were estimated using lifetime data based on the Kaplan–Meier method, with 95% confidence intervals (CIs). For evaluating success rates, patients lost to follow‐up at 10 years were excluded. Success rates were calculated at each follow‐up visit by dividing the number of patients meeting all success criteria by the total patient population at that visit.

At each follow‐up visit, the average bone level was subtracted from the average baseline bone level to determine the mean bone level change. A positive change indicated bone gain from baseline to follow‐up, while a negative change indicated bone loss. Missing values in the full‐analysis population with imputation were replaced using appropriate imputation methods (mean values of the remaining patients for the specific measurement and time point). Sensitivity analysis was conducted by repeating the analyses without imputing missing values. As previously described, descriptive statistics were calculated for all documented parameters, including means and standard deviations. Where applicable, 95% confidence intervals were provided to support the descriptive data. Paired t‐tests were used to assess the significance of changes in bone levels at baseline, and at 0.5, 1, 2, 3, 5, and 10 years following implant placement. Further details regarding the statistical analysis can be found in the previous publications reporting on this study population (Bormann et al. 2018; Gahlert et al. 2022, 2016).

## 3 Results

This paper only presents the clinical and radiographic data collected after 10 years of follow‐up. Data from the 1‐, 3‐, and 5‐year follow‐ups have been published previously (Bormann et al. 2018, Gahlert et al. 2022, 2016).

### 3.1 Patients

Between October 2011 and July 2012, 44 patients (27 females and 17 males) underwent a total of 44 implant placements as part of this study. At the time of implant placement, the mean age of the patients was 48 ± 14 years (range: 18 to 78 years). Bone augmentation prior to implant placement was necessary in 3 patients (6.8%), while 14 patients (31.8%) required osseous augmentative procedures during implant placement. The most common indication for bone augmentation was a vestibular and/or central bone deficiency (*n* = 8, 57.1%), followed by alveolar deficits (*n* = 4, 28.6%) and buccal dehiscence (*n* = 2, 14.3%).

During follow‐up, 3 patients were lost to follow‐up within the first 3 years, 2 more discontinued participation before the 5‐year visit, and another 4 dropped out between years 5 and 10. Specifically, 5 patients discontinued participation before the 5‐year follow‐up for non‐implant–related reasons (e.g., relocation, general health issues), and 4 patients were lost to follow‐up between years 5 and 10. Importantly, none of these patients experienced implant loss before discontinuation, and no implants were explanted in this group. Thus, the only documented implant loss occurred in a patient who remained under observation and was included in the analysis. By December 2022, the last patient completed the 10‐year follow‐up. Consequently, data from 35 patients (21 females and 14 males; mean age: 57 ± 14 years, range: 29–86 years) with 35 implants were available for final analysis.

The distribution of implants among the investigated patients was relatively balanced, with 19 implants (54.3%) placed in the anterior region and 13 implants (37.1%) inserted in the posterior maxilla. In contrast, only 3 implants (8.6%) were placed in the posterior mandible (Table 1).

**TABLE 1:** Distribution of implants placed in the 10‐year follow‐up population.

	*n*	[%]	Posterior [*n*]				Anterior [*n*]			Anterior [*n*]			Posterior [*n*]			
			8				11			8			5			
Maxilla	32	91.4	19							13						
Number of implants			0	3	1	4	0	5	6	5	3	0	4	1	0	0
Implant Location^a^			17	16	15	14	13	12	11	21	22	23	24	25	26	27
Implant Location^a^			47	46	45	44	43	42	41	31	32	33	34	35	36	37
Number of implants			0	2	0	1	0	0	0	0	0	0	0	0	0	0
Mandibula	3	8.6	3							0						
			3				0			0			0			
Total	35	100	posterior [*n*]				anterior [*n*]			anterior [*n*]			posterior [*n*]			

Abbreviation: *n*, number.

^a^ Implant position according to FDI.

### 3.2 Implant Survival

**FIGURE 1:**
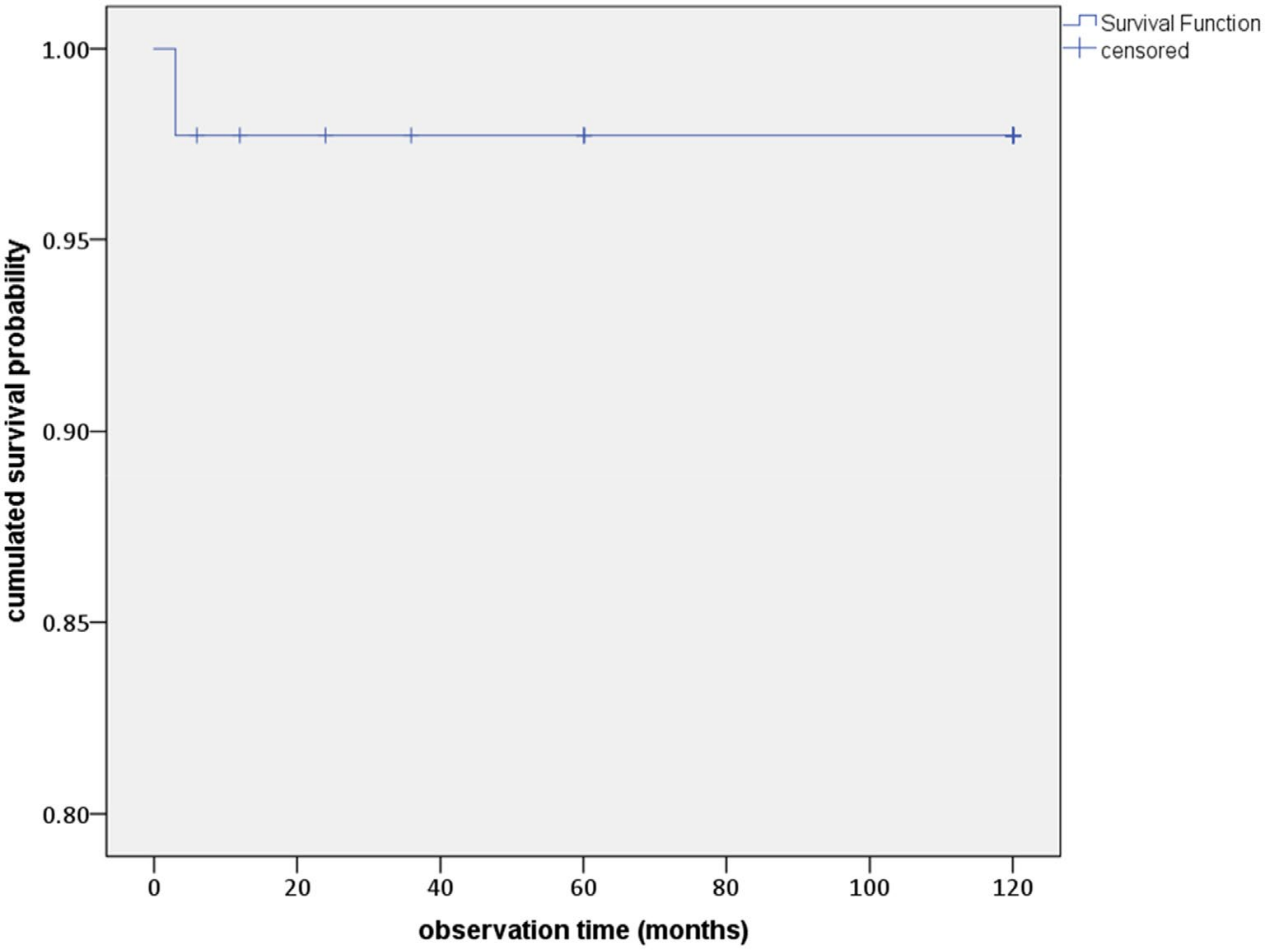
Survival rate of investigated implants over 10 years (Kaplan–Meier analysis).

Between implant placement and the last follow‐up investigation at 10 years, only 1 out of the 35 implants was lost prior to loading, resulting in one early failure. This lost implant was located in the anterior maxilla (position 21 according to FDI). The estimated survival rate at 10 years after implant placement, calculated using the Kaplan–Meier method, was 97.7% (95% CI: 97.27–98.13, Figure 1).

### 3.3 Implant Success

At the 10‐year follow‐up, 3 implants (8.6%) exhibited biological complications and did not meet the success criteria. Specifically, 2 implants (5.7%) were diagnosed with peri‐implant mucositis, while 1 implant (2.9%) presented with peri‐implantitis accompanied by continuous radiolucency around the implant. Notably, all biological complications occurred in the anterior maxilla (position 21 according to FDI) and showed no association with specific patient‐related factors such as smoking, poor oral hygiene, systemic diseases, or medication intake. As a result, the evaluated success rate for the investigated implants was 91.4% (95% CI: 76.9–97.8).

### 3.4 Radiographic Examination

**FIGURE 2:**
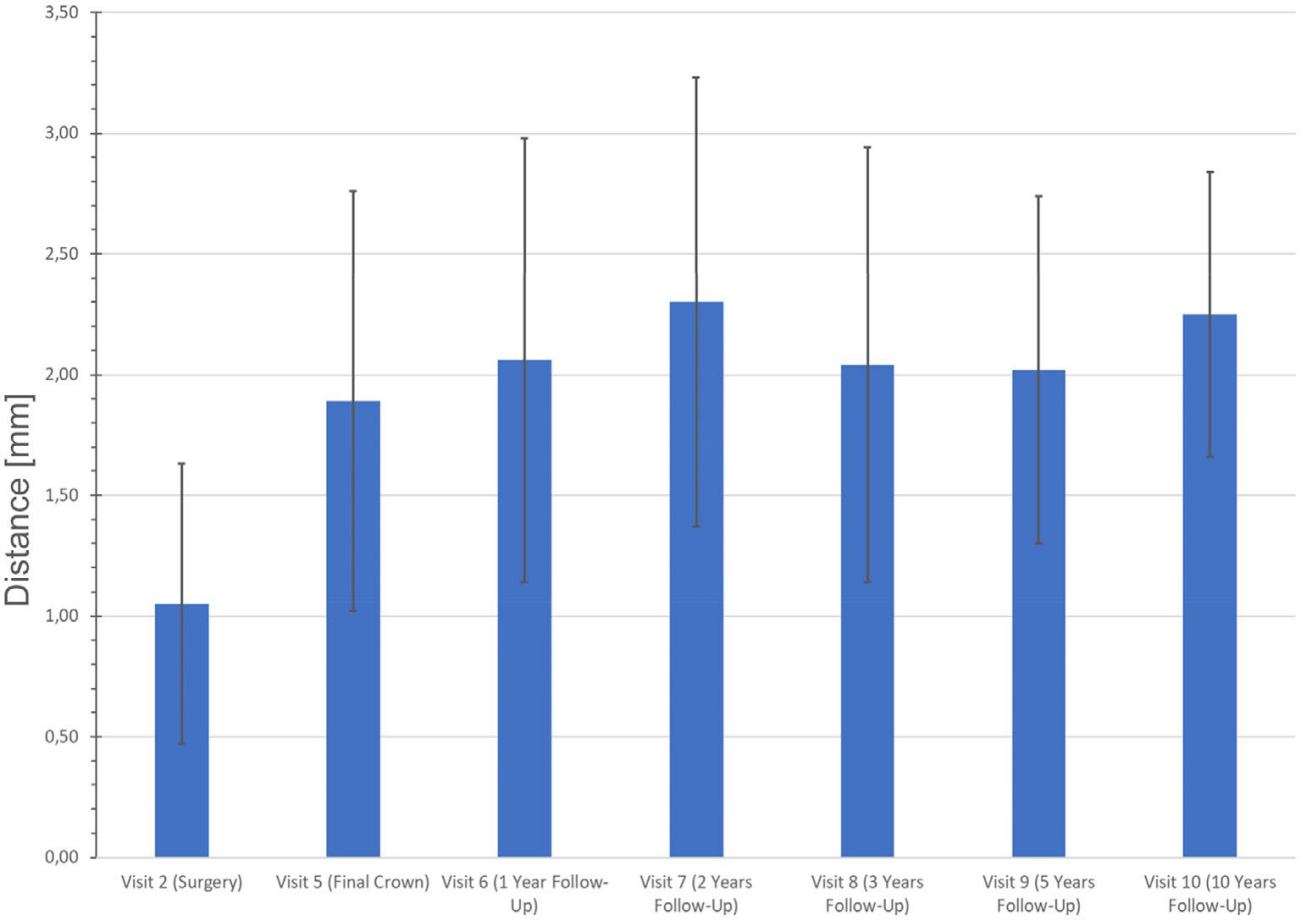
Peri‐implant bone level changes (mean ± SD) in mm from implant placement over each follow‐up time point up to 10 years.

Peri‐implant bone levels were assessed in the patient population (*n* = 35). The distance from the implant shoulder to the first visible bone‐to‐implant contact (DIB) measured immediately after implant placement ranged from 0.0 mm to 2.47 mm, yielding a mean DIB_0_ value of 1.05 ± 0.58 mm, which indicates a slightly subcrestal position of the implant shoulder. At the last follow‐up, the mean DIB_6_ value was 2.25 ± 0.59 mm (with a range of 1.37 mm to 4.52 mm, Table 2, Figure 2).

**TABLE 2:** Peri‐implant bone levels in mm (mean ± SD) from implant surgery to 10‐year follow‐up visit.

Radiographic parameters	*N*	Mean	Standard	Range
DIB_0_	35	1.05	0.58	0.00–2.47
DIB_0.5_	35	1.89	0.87	0.28–5.10
DIB_1_	35	2.06	0.92	0.26–5.25
DIB_2_	35	2.30	0.93	0.31–5.37
DIB_3_	35	2.04	0.90	0.32–4.37
DIB_5_	35	2.02	0.72	0.43–3.60
DIB_10_	35	2.25	0.59	1.37–4.52
DIB_0_‐DIB_0.5_ *	35	−0.84	0.77	−4.44 to −0.07
DIB_0_‐DIB_1_ *	35	−1.00	0.83	−4.58 to 0.17
DIB_0_‐DIB_2_ *	35	−1.25	0.89	−4.71 to 0.22
DIB_0_‐DIB_3_ *	35	−0.98	0.84	−3.02 to 0.76
DIB_0_‐DIB_5_ *	35	−0.97	0.54	−2.28 to 0.36
DIB_0_‐DIB_10_ *	35	−1.20	0.61	−2.82 to −0.02

* Statistically significant difference (*p* < 0.001).

**FIGURE 3:**
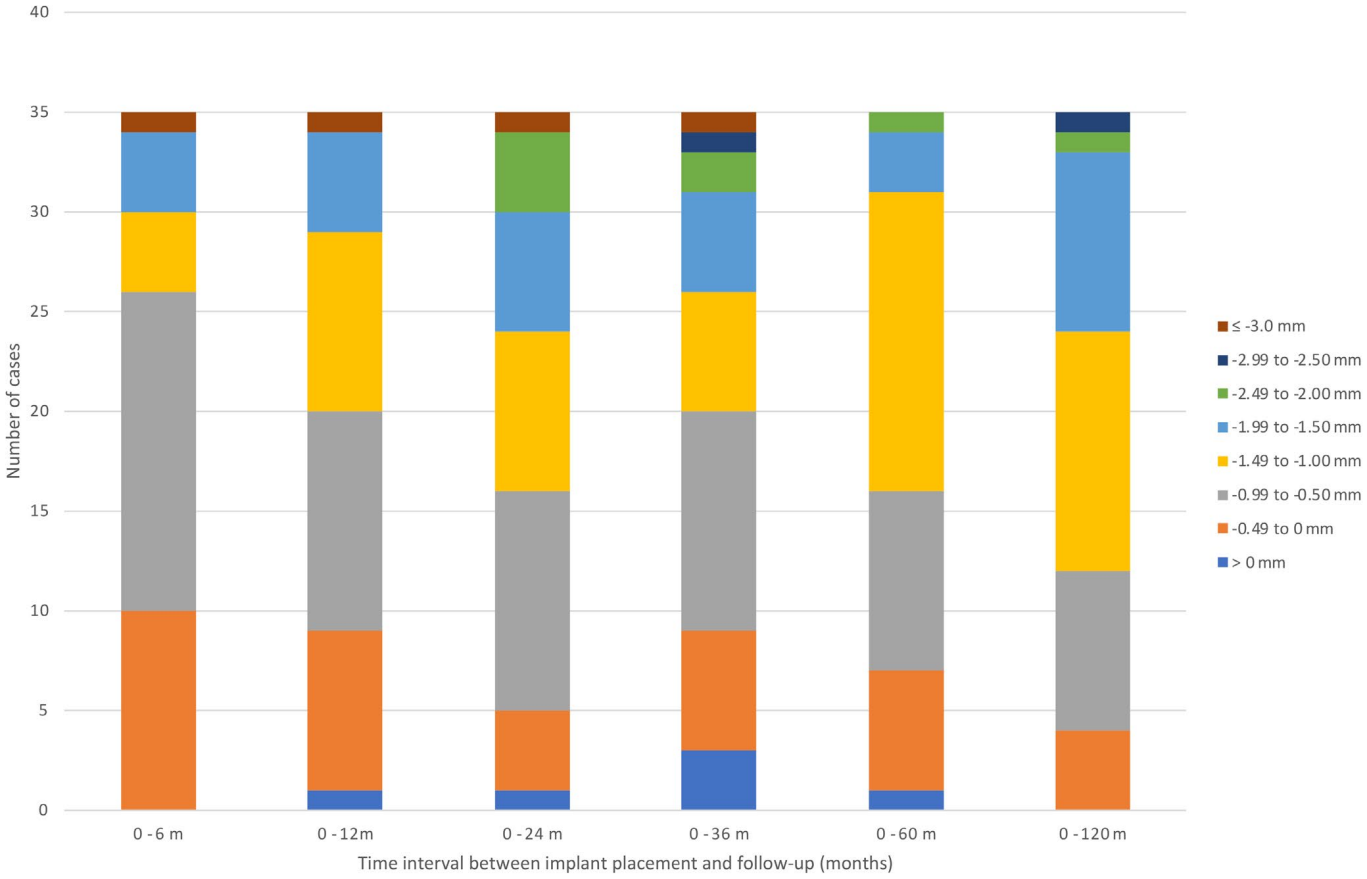
Distribution of peri‐implant bone level changes over 10 years.

**FIGURE 4:**
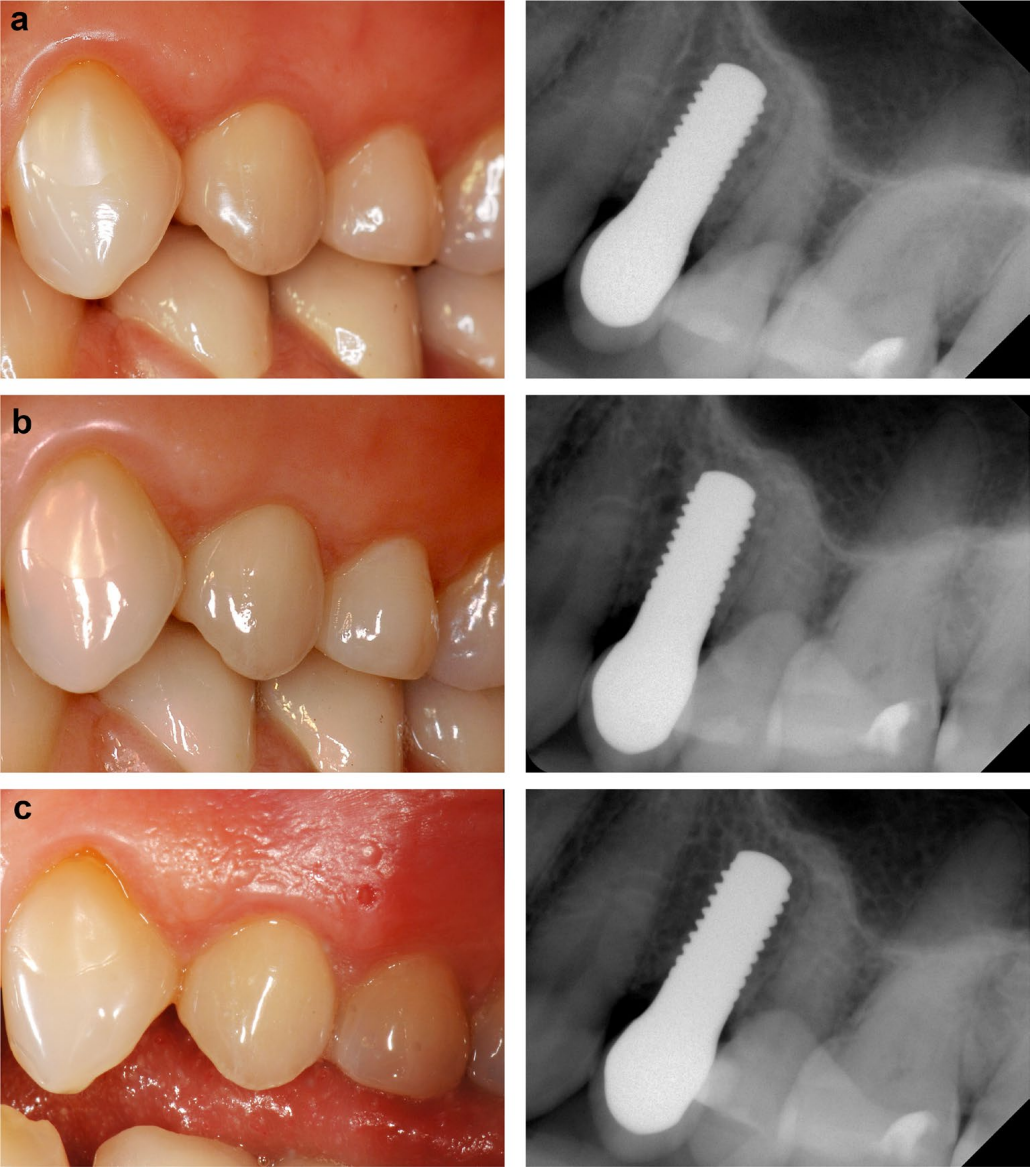
Clinical case at 1 year (a), 5 years (b) and 10 years (c) after implant placement.

**FIGURE 5:**
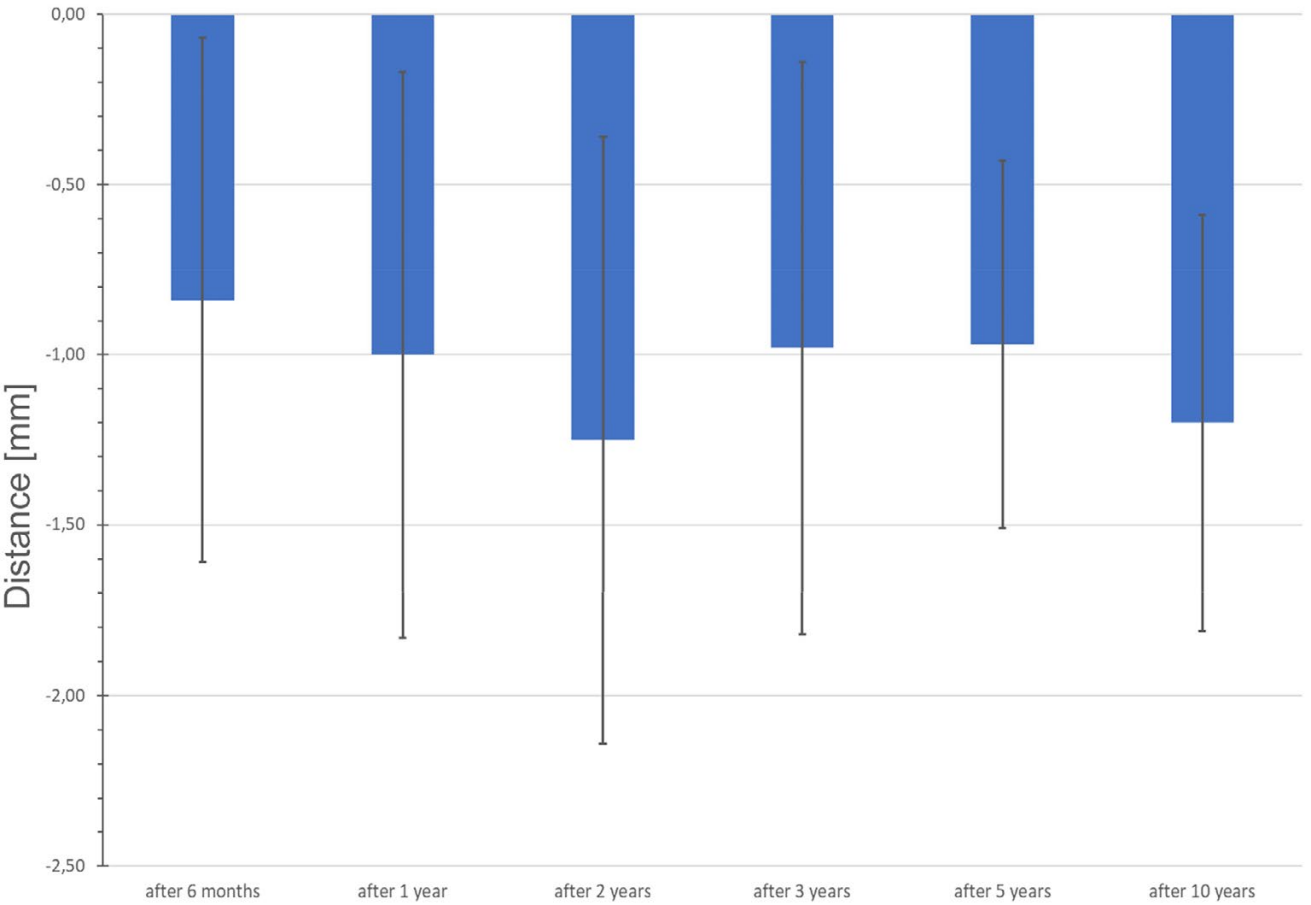
Peri‐implant bone levels in mm (mean ± SD) from implant placement over each follow‐up time point up to 10 years.

The analysis of peri‐implant bone level changes over the 10‐year observation period reveals that the most significant bone remodeling occurred within the first 2 years following implant placement, with a peak bone loss of −1.25 mm ± 0.89 mm (DIB_0_ –DIB_2_). Most implants showed changes between −1.50 mm and −0.50 mm at all time points, reflecting typical early remodeling. In the first 24 months, a shift toward more negative values was observed, indicating initial bone adaptation. After 36 months, the distribution stabilized, with no increase in severe bone‐loss categories and only isolated cases showing slight bone gain during the early phase. However, no implants exhibited radiographically measurable bone gain after 10 years, supporting the pattern that most bone remodeling occurred early, followed by long‐term stabilization (Figure 3). Interestingly, a slight decrease in the DIB values between years 2 and 5 (DIB_2_ to DIB_3_ and DIB_5_) was observed, suggesting a possible phase of bone stabilization or even minor osseous regeneration around the implants. Following this intermediate period, bone levels remained largely stable, with only a mild increase in bone loss observed by year 10. This results in a total mean bone loss of −1.20 mm ± 0.61 mm compared to baseline (DIB_0_—DIB_10_), indicating moderate peri‐implant bone loss of approximately 1.2 mm over the entire study period (Figure 2). Figure 4 clearly demonstrates stable peri‐implant bone levels over the 10‐year period, reinforcing the overall findings. All peri‐implant bone level changes compared to baseline were statistically significant (*p* < 0.001, Table 2; Figure 5).

### 3.5 Clinical Examination

**FIGURE 6:**
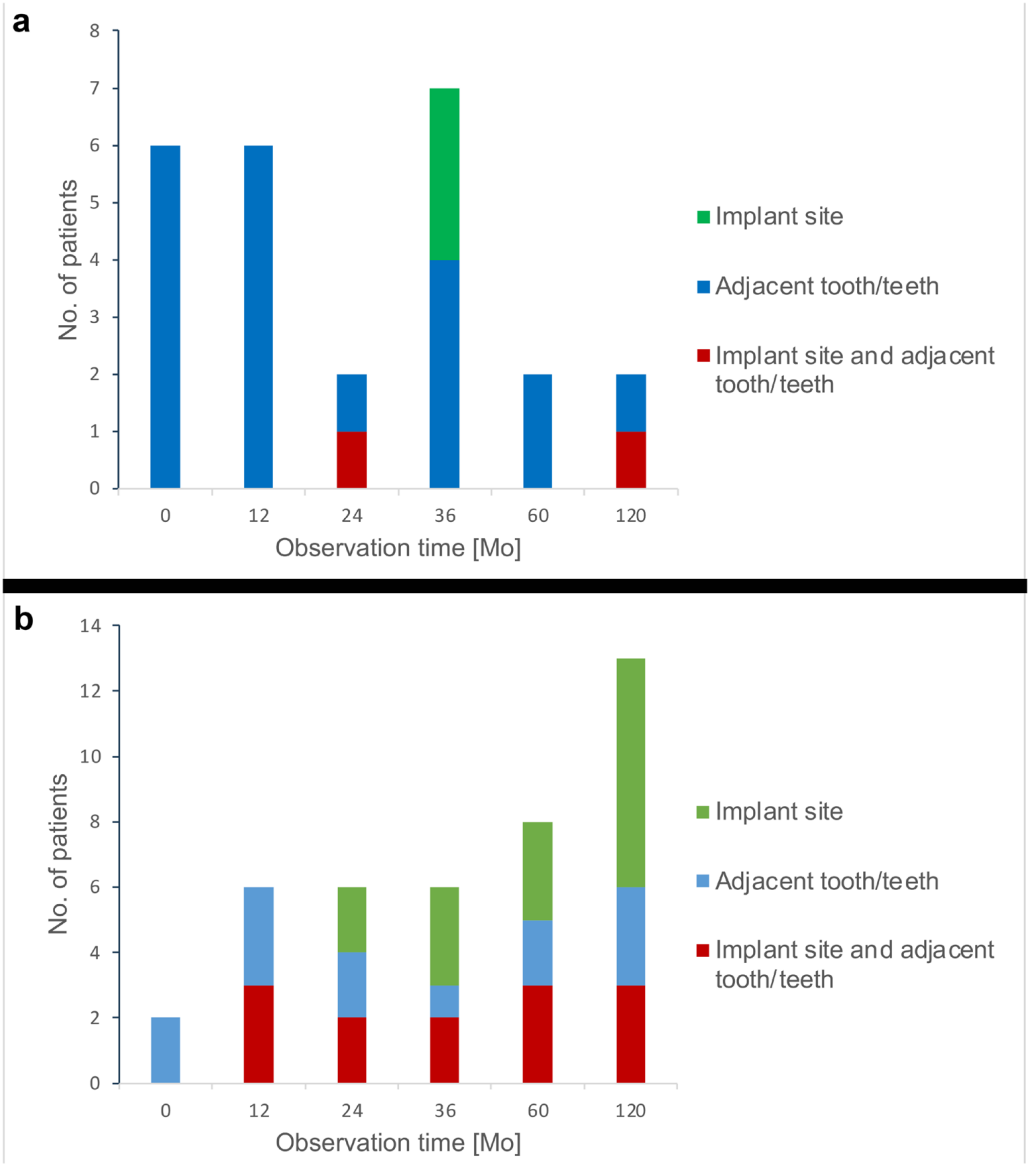
Location of plaque (a) and bleeding occurrence (b).

During the follow‐up, plaque was observed in 12 patients (34.3%), primarily during the initial screening (*n* = 6, 50%), and again at the 1‐year (*n* = 6, 50%) and 3‐year (*n* = 7, 58.3%) follow‐ups. In most cases, plaque was consistently detected on the adjacent teeth. Only at the 3‐year follow‐up were 3 occurrences (25%) noted at the implant site. After 10 years, plaque was present in 2 patients: in 1 patient (8.3%) at the adjacent teeth and in another patient (8.3%) at both the implant site and adjacent teeth, with no plaque observed solely at the implant sites (Figure 6).

Soft tissue bleeding was detected at the adjacent teeth or implant site in 18 patients (51.4%), with the highest incidence observed during the 5‐year (*n* = 8, 44.4%) and 10‐year (*n* = 13, 72.2%) follow‐ups. At the 10‐year follow‐up, bleeding was noted at 7 implant sites, at 3 adjacent teeth, and at both implant sites and adjacent teeth (Figure 6). Importantly, this soft tissue bleeding was not associated with peri‐implantitis.

Adverse events (AEs) up to 5 years of follow‐up have been previously reported (Bormann et al. 2018; Gahlert et al. 2022). Between the 5‐ and 10‐year follow‐up, 8 AEs (22.9%) were observed in 3 patients. Four AEs (11.4%) were deemed unrelated to the study device, including the extraction of another tooth, additional implant placement, occurrence of periodontitis, and root canal treatment (*n* = 1, 2.9% each). Four AEs (11.4%) were related to the study device: chipping of the ceramic crown (*n* = 1, 2.9%), peri‐implant mucositis (*n* = 2, 5.7%), and peri‐implantitis (*n* = 1, 2.9%).

### 3.6 Esthetic Outcomes

The PES/WES scores after 5 and 10 years were similar, with mean values of 14.5 for both time points (5 years: SD ±2.1, range 9–18; 10 years: SD ±2.4, range 10–18). For the PES, the mean values increased from 7.4 (±1.7, range 4–10) at 5 years to 7.8 (±1.6, range 5–10) at 10 years. After 5 years, the parameters with the highest mean values were the mesial papilla (1.6 ± 0.5) and facial mucosa curvature (1.6 ± 0.5). After 10 years, the highest values were recorded for the mesial (1.8 ± 0.4) and distal papilla (1.7 ± 0.7).

In both investigation periods, the combined variable of root convexity, soft tissue color, and texture showed the lowest mean values of 1.3 (5 years: SD ± 0.5; 10 years: SD ± 0.5). Figure 4 demonstrates that the peri‐implant mucosa remains stable over the 10‐year period, which underscores the increase in PES scores during this time.

Regarding the WES, the mean scores decreased from 7.0 (±1.4) after 5 years to 6.7 (±1.6) after 10 years. The parameters with the highest scores were tooth form (5 years: 1.6 ± 0.5; 10 years: 1.5 ± 0.5) and the outline and volume of the clinical crown (5 years: 1.8 ± 0.4; 10 years: 1.6 ± 0.6; Table 3).

**TABLE 3:** PES/WES evaluation after 5 and 10 years.

	Pink Esthetic Score PES					Total PES	White Esthetic Score WES					Total WES	Total PES & WES
	Mesial papilla	Distal papilla	Curvature labial mucosa	Level facial mucosa	Root convexity, soft tissue color and texture		Tooth form	Outline and volume of the clinical crown	Color which indicates the assessment of the dimension's hue and value	Surface texture	Translucency and characterization		
10 years													
Mean	1.8	1.7	1.6	1.5	1.3	7.8	1.5	1.6	1.3	1.2	1.2	6.7	14.5
SD	0.4	0.7	0.5	0.6	0.5	1.6	0.5	0.6	0.5	0.5	0.5	1.6	2.4
Min	1	0	1	0	1	5.0	1	0	1	0	0	4	10
Max	2	2	2	2	2	10.0	2	2	2	2	2	10	18
5 years													
Mean	1.6	1.5	1.6	1.4	1.3	7.4	1.6	1.8	1.4	1.3	1.1	7.0	14.5
SD	0.5	0.6	0.5	0.7	0.5	1.7	0.5	0.4	0.5	0.4	0.3	1.4	2.1
Min	1	0	1	0	1	4.0	1	1	1	1	1	5	9
Max	2	2	2	2	2	10.0	2	2	2	2	2	10	18

## 4 Discussion

In the present multicenter prospective study, the clinical performance of a commercially available 1‐piece zirconia dental implant was investigated. After 10 years of functional loading, the implants demonstrated high survival and success rates comparable to those of commonly used titanium implants, with few technical and biological complications, minimal peri‐implant marginal bone loss, and esthetic outcomes with clinically acceptable ranges.

Existing literature shows that the physical properties and ongoing market availability of zirconia implants influence survival. A meta‐analysis reported a 1‐year survival of 98.3% for commercially available zirconia implants versus 91.2% for systems no longer marketed, and fracture incidence decreased from 3.4% in 2004 to 0.2% in 2017 (Roehling et al. 2018). Since the currently investigated implant type is commercially available and has been evaluated in the same patient population at 1, 3, and 5 years (Bormann et al. 2018; Gahlert et al. 2022, 2016), the present 10‐year results are clinically relevant.

In this study, only one of 35 implants was lost before loading, resulting in an estimated 10‐year survival of 97.7% (95% CI: 97.27–98.13), which is comparable to the 95.1%–98.9% survival rates reported for titanium implants after 10 years (Buser et al. 2012; Di Francesco et al. 2022; Fischer and Stenberg 2012; Kim et al. 2018).

Regarding the clinical performance of zirconia implants, a meta‐analysis evaluating 277 commercially available zirconia implants placed in 221 patients estimated a mean 5‐year survival rate of 97.2% (95% CI 94.7–99.1) (Roehling et al. 2023). Only a few studies have extended follow‐up beyond 5 years. The reported survival rates range from 93.8% to 100%, with small prospective and retrospective cohorts of 1‐ and 2‐piece systems (Borgonovo et al. 2021; Brunello et al. 2022; Karapataki et al. 2023; Koller et al. 2020). Importantly, none of these long‐term studies reported implant fractures.

While the present study demonstrated a high long‐term survival rate for 1‐piece zirconia implants, it is important to consider factors that have been associated with early implant failure in the broader literature. Several implant‐related variables—such as tapered implant design, short implant lengths (< 10 mm), non‐submerged healing, low insertion torque (< 30 Ncm), and Type I bone quality—have been identified as significant predictors of early failure in titanium implant systems (Dong et al. 2025; Lin et al. 2025; Yari et al. 2024). Conical and bone‐level titanium implants show higher early failure than cylindrical or tissue‐level designs, which is relevant since the implant examined in the current study was a 1‐piece, tissue‐level zirconia with non‐submerged healing. Current data on zirconia implants remain limited, with only 1 study comparing 1 and 2‐piece designs and finding no survival difference (Roehling et al. 2018). Additionally, while the present cohort included a variety of reasons for tooth loss, previous research has shown that this factor does not significantly influence early implant failure (Dong et al. 2025), highlighting the need for context‐specific evaluation of zirconia implant outcomes.

Between the 5‐ and 10‐year follow‐ups, 8 additional adverse events were recorded, 4 related to the study device. Three were biological complications (8.6%), lowering the success rate from 97.2% (95% CI: 84.6–99.9) to 91.4% (95% CI: 0.77–0.98). All complications, including crown chipping, were successfully treated, but the decline highlights the impact of extended follow‐up on implant success.

The evaluated success rate in the present study is lower than that reported for titanium implants, which showed a success rate of 97.0% after 10 years (Buser et al. 2012). However, direct comparison between the two studies is not clinically meaningful due to substantial differences in study design and the type of implants investigated. Buser et al. conducted a retrospective analysis of 511 titanium implants, while the current study prospectively evaluated only 35 implants. In addition, the present study considered a broader range of clinical factors, which may also have contributed to the slightly lower overall success rate.

Bone level analysis showed mean marginal bone loss of 1.20 mm ± 0.61 after 10 years. These results align with previously reported data on titanium and zirconia implants at similar time points (Borgonovo et al. 2021; Buser et al. 2012; Fischer and Stenberg 2012; Koller et al. 2020). At the time of implant placement, the distance between the implant shoulder and the first visible bone‐to‐implant contact (DIB_0_) ranged from 0.0 to 2.47 mm. Notably, some implants exhibited higher DIB values at baseline (DIB_0_ up to 2.47 mm) and during early follow‐up (DIB_2_ up to 5.37 mm). Since the polished collar measures 1.8 mm, this variation indicates that some implants were placed deeper (subcrestal) and others higher (supracrestal) than the intended position. This explains both the higher and lower baseline values. In subcrestally placed implants, greater early marginal bone remodeling occurred, as the bone level adapted to the transition between the smooth and micro‐rough surface. Conversely, in supracrestally positioned implants, a reduction of the DIB was observed in some cases most likely due to a “creeping attachment” phenomenon of the bone, which explains the isolated instances of apparent bone gain.

Most implants showed changes between −1.50 mm and −0.50 mm at all time points, reflecting typical early remodeling. A shift toward more negative values occurred within the first 24 months, followed by stabilization after 36 months, with no increase in severe bone‐loss categories and only isolated early cases of slight bone gain. No implants showed radiographically measurable bone gain at 10 years, confirming that most remodeling occurred early, followed by long‐term stability.

In terms of progression, the marginal bone loss between placement and the final follow‐up was 0.99 mm ± 0.58 after 5 years and 1.20 mm ± 0.61 after 10 years. This indicates that 82.5% of the peri‐implant marginal bone loss occurred within the first 5 years, with only minor additional changes thereafter, reflecting a stabilization of bone levels over the long term.

The decrease in marginal bone loss over time suggests that peri‐implant bone levels stabilized after the initial remodeling phase. This early remodeling, linked to subcrestal placement and functional loading, reflects typical healing and adaptation processes. The subsequent stability over the long‐term supports the favorable prognosis of zirconia implants under functional conditions.

In addition, esthetic outcomes were assessed using the PES/WES scores. By including PES and WES, the present study provides valuable insights into the soft tissue integration and visual outcomes of zirconia implants from a patient‐centered perspective. Moreover, the use of these standardized indices allows for direct comparison with other studies that have assessed esthetic outcomes using the same criteria, thereby enhancing the scientific relevance and external validity of the findings. The presented data on the PES/WES after 5 and 10 years provide critical insights into the performance of zirconia implants, particularly in terms of soft tissue integration and the esthetic appearance of the restorations. The increase in the mean PES from 7.4 at 5 years to 7.8 at 10 years suggests an enhancement in soft tissue parameters over time. This improvement is particularly notable for the mesial papilla and facial mucosa curvature, which exhibited the highest mean values after both time periods. These findings underscore the importance of soft tissue management and highlight the excellent esthetic properties of ceramic implants, especially regarding soft tissue esthetic outcomes. In contrast, the WES revealed a decline from a mean of 7.0 at 5 years to 6.7 at 10 years, indicating a perceived reduction in esthetic parameters associated with ceramic implant‐supported restorations over time. Regarding the esthetic outcomes of titanium implants, studies have reported similar values for PES (ranging from 7.0 to 8.3), WES (ranging from 6.3 to 7.9), and combined PES/WES scores (ranging from 7.1 to 14.6) at comparable follow‐up periods (Amorfini et al. 2018; Bonde et al. 2013; Fonseca et al. 2021). To date, only a few clinical studies have examined the PES score around zirconia implants over time. Koller et al. reported a slight PES decrease for zirconia (11.4 to 11.1) and an increase for titanium (11.1 to 11.6) after 30–80 months, though the cohort was small (15 zirconia and 16 titanium implants) (Koller et al. 2020). In addition, a randomized trial with 30 patients found stable and comparable PES values for 1‐piece zirconia and titanium tissue‐level implants over 5 years, with only slight decreases (zirconia: 7.81 to 7.44; titanium: 7.86 to 7.43) (Ruiz Henao et al. 2021, 2024). These results further support the long‐term esthetic predictability and reliability of 1‐piece zirconia implants.

Despite the encouraging results, several limitations must be acknowledged. Most notably, the relatively small sample size of 35 implants limits the statistical power of the findings and restricts their generalizability. Additionally, the study design did not include a direct control group with titanium implants, which precludes direct material‐based comparisons. Although the multicenter and prospective approach adds robustness, variations in clinical handling across centers could introduce subtle inconsistencies. Furthermore, the absence of randomization and the specific inclusion criteria may have introduced potential selection bias, which could influence the external validity of the results. Another important aspect to consider is the low number of implants placed in the mandible, which may have affected the outcomes. Finally, the dropout rate of 20.5% over 10 years represents an additional limitation; while no implant loss was documented within this group, MNAR (missing not at random) cannot be fully excluded. Nevertheless, the dropout rate is comparable—or even lower—than those reported in other 10‐year implant studies (Gadzo et al. 2023; Roccuzzo et al. 2020; van Velzen et al. 2015). Moreover, survival estimates were derived using the Kaplan–Meier method, which assumes non‐informative censoring. While none of the patients lost to follow‐up experienced implant failure before dropout, the possibility that censoring was related to unobserved complications cannot be completely excluded. With respect to radiographic measurements, it should also be noted that, despite standardized assessment procedures, minor variability inherent to repeated DIB measurements cannot be entirely ruled out. Future studies with larger cohorts and standardized protocols are needed to validate and expand upon these long‐term results.

## 5 Conclusion

This prospective multicenter study is the first to evaluate commercially available 1‐piece zirconia implants over a 10‐year period. The implants demonstrated a high survival rate (97.7%), moderate bone loss (1.20 mm), and stable esthetic outcomes. These results confirm that zirconia implants offer clinical reliability and esthetic performance comparable to titanium, establishing them as an equivalent and viable long‐term alternative. However, interpretation is limited by the small sample size, lack of a control group, potential selection bias, and inter‐center variability.

## Author Contributions

**S. Roehling:** led the conceptualization, investigation, and validation of the study. He contributed to data collection and analysis, managed the project, and played a central role in writing and editing the manuscript. **K. H. Bormann:** contributed to the study's conceptualization and validation, participated in the investigation, and worked closely with S. Roehling on writing and editing the manuscript. **M. M. Bornstein:** focused on the investigation and analysis phase, contributing to data collection and analysis. He also played a significant role in writing the original draft and manuscript revisions. **S. Laval:** contributed to the investigation, assisting with data collection, and supported the writing of the original draft by providing key input. **F. Thieringer:** supervised the study, guided the investigation, and contributed to the writing and editing of the manuscript. **M. Gahlert:** as principal investigator of the study, he was involved in conceptualization, project administration, and data analysis. He contributed to writing and editing the manuscript and ensured the study's successful execution.

## Conflicts of Interest

The authors declare no conflicts of interest.

## Supporting information


**Data S1:** clr70089‐sup‐0001‐Supinfo.docx.

## Data Availability

The data that support the findings of this study are available on request from the corresponding author. The data are not publicly available due to privacy or ethical restrictions.
